# Self-rated Health and Related Factors before and during the COVID-19 Outbreak in Japan

**DOI:** 10.31662/jmaj.2022-0171

**Published:** 2023-03-13

**Authors:** Haruka Ishii, Yoko Ishii

**Affiliations:** 1 Toyama Prefectural Central Hospital, Toyama, Japan; 2 Graduate School of Health and Nutrition Sciences, The University of Nagano, Nagano, Japan

**Keywords:** self-rated health, COVID-19, subjective economic state, healthy behaviors

## Introduction

Self-rated health (SRH) status is correlated with objective health indicators ^(1)^. It is a good predictor of morbidity, mortality, and other health outcomes ^(2), (3)^. Moreover, it has been effectively used in studies to document social inequalities in health ^(4), (5)^. The coronavirus disease 2019 (COVID-19) outbreak measures have had a large impact on people’s lives. In Japan, the government declared a state of emergency in seven prefectures on April 07, 2020. A nationwide emergency was imposed on 16 April 2020 and ended on May 25, 2020. This study aimed to compare the factors related to SRH before and during the spread of COVID-19. To reveal how SRH changed during COVID-19 outbreak in Japan, we examined SRH change before and during the COVID-19 outbreak by a secondary analysis of the data in the “Survey of Public Awareness for Food and Nutrition Education” conducted by the Cabinet Office of Japan in 2018, 2019, and 2020.

## Materials and Methods

We used individual data from the three independent “Surveys of Attitudes toward Food and Nutrition Education,” conducted by the Cabinet Office of Japan in 2018, 2019, and 2020 (S-2018, S-2019, and S-2020). The three surveys were performed in October 2018, November/December 2019, and December 2020. We used the data of S-2018-2019 and S-2020 as those before and during COVID-19 outbreak, respectively.

For each nationwide survey, a two-stage stratified random sampling was used to select 3,000 people aged 20 or older. The survey sample was representative of the Japanese population and accounted for regional differences across the country, and the number of people surveyed in each municipality was proportionate to the municipality’s population. Therefore, we assessed that the survey sample was appropriate to estimate SRH before and during COVID-19 outbreak. There were 1824, 1721, and 2395 participants for S-2018, S-2019, and S-2020, respectively. After removing missing data, 1823, 1709, and 2364 respondents provided adequate data for further analyses.

We used the following questions, all of which were the same for the S-2018, S-2019, and S-2020. SRH was assessed based on the question: “How would you rate your general health status?.” The responses were rated on a five-point Likert scale (“good,” “fairly good,” “neither good nor bad,” “fairly bad,” and “bad”). Subjective economic status (SES) was assessed based on the question: “How would you rate your economic status?.” The responses were rated on a five-point Likert scale (“good,” “fairly good,” “neither good nor bad,” “fairly bad,” and “bad”). Healthy behaviors for prevention of lifestyle diseases were assessed based on the question: “For the prevention and improvement of lifestyle diseases, to what extent do you behave? For example, weight management, reduced salt, and so on.” The responses were rated on a five-point Likert scale (“usually,” “often,” “rarely,” “never,” and “do not know”). Residential area was categorized into five city sizes: big city, medium-sized cities (cities with a population of 200,000 or more), medium-sized cities (cities with a population of 100,000 or more), small cities (cities with less than 100,000 inhabitants), and towns and villages.

To analyze the differences in the distribution of the items (gender, SRH, SES, healthy behaviors, and city size) among the S-2018, S-2019, and S-2020, the chi-square test was used. In addition, to analyze differences in SRH, SES, and healthy behaviors among S-2018, S-2019, and S-2020, nonparametric tests (the Kruskal-Wallis analysis and Bonferroni’s multiple comparison test) were conducted because the data were nonnormally distributed. Binomial logistic regression models were used to obtain odds ratios (OR) and their 95% confidence intervals for SES and healthy behaviors as predictors of SRH, after adjusting for age, gender, and residential areas.

All data were analyzed using SPSS version 28.0 (IBM Japan, Ltd., Tokyo, Japan), with a significance level of 0.05.

Ethics approval was not required for this secondary analysis of data publicly available through the University of Tokyo’s Center for Social Research and Data Archives, Institute of Social Science.

## Results

**Figure 1.**
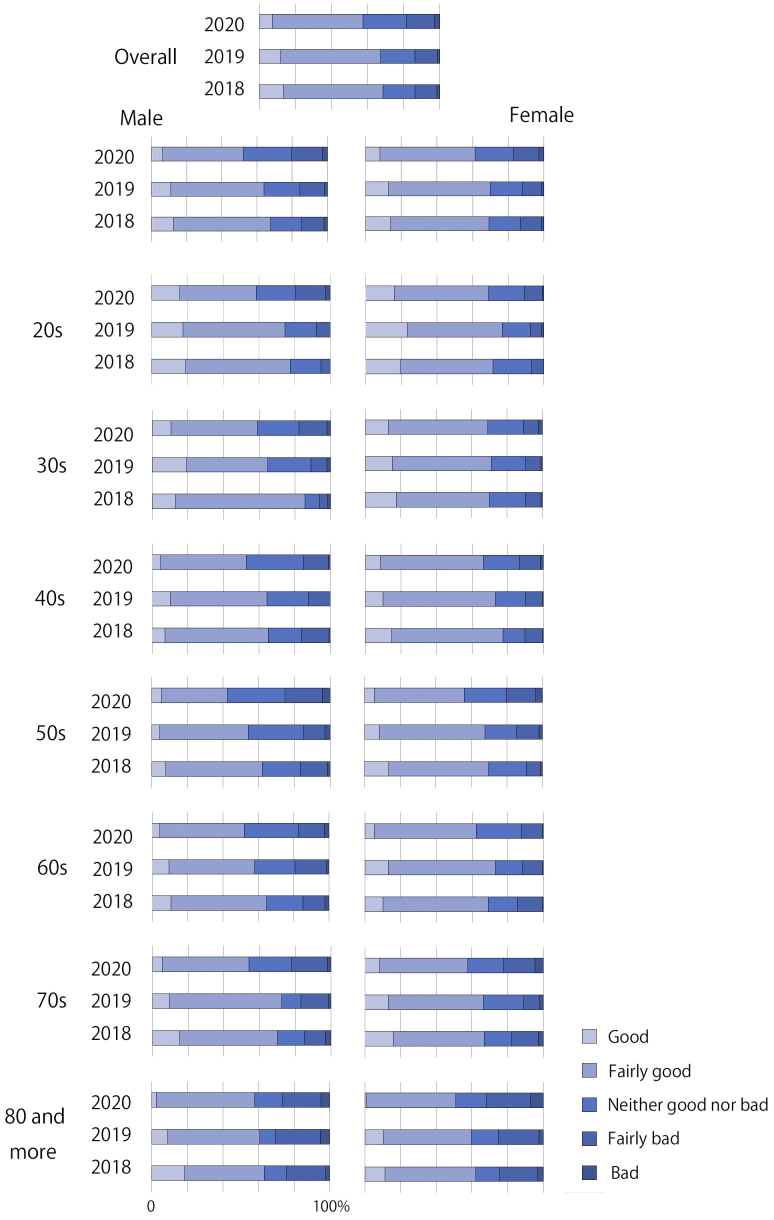
Percentage of self-rated health (SRH) category.

Table 1 presents the demographic characteristics of the participants of S-2018, S-2019, and S-2020. The mean age of the participants of S-2018, S-2019, and S-2020 was 56.8 ± 16.6, 56.1 ± 17.5, and 56.8 ± 17.1 years, respectively. The median age of those was 58, 57, and 58 years, respectively. Figure 1 shows the percentage of SRH category. In S-2020, the percentages of good/fairly good SRH decreased in both male and female in all ages compared with those in S-2018 and S-2019.

**Table 1.** Characteristics of Participants and Univariate Analysis.

Variable	S-2018		S-2019		S-2020		P1	P2	P3
n, overall	1824		1721		2395				
Age, mean SD	56.8, 16.6		56.1, 17.5		56.8, 17.1				
n, male (%)	799	(42.8)	759	(44.1)	1032	(43.1)	0.794		
n, female (%)	1025	(56.2)	962	(55.9)	1363	(56.9)			
Self-rated health (SRH) (%)									
Very good	242	(13.3)	201	(11.8)	169	(7.1)	<0.001	<0.001	
Fairly good	1007	(55.2)	948	(55.5)	1192	(50.4)			S-2018 vs. S-2019: 0.926
Neither good nor bad	321	(17.6)	327	(19.1)	567	(24.0)			S-2018 vs. S-2020: <0.001
Fairly bad	225	(12.3)	209	(12.2)	369	(15.6)			S-2019 vs. S-2020: <0.001
Bad	28	(1.5)	24	(1.4)	67	(2.8)			
Subjective economic status (SES) (%)									
Very good	190	(10.4)	236	(13.8)	202	(8.5)	<0.001	<0.001	
Fairly good	684	(37.5)	569	(33.3)	747	(31.6)			S-2018 vs. S-2019: 0.855
Neither good nor bad	604	(33.1)	538	(31.5)	758	(32.1)			S-2018 vs. S-2020: <0.001
Fairly bad	291	(16.0)	314	(18.4)	516	(21.8)			S-2019 vs. S-2020: <0.001
Bad	54	(3.0)	52	(3.0)	141	(6.0)			
Healthy behaviors for preventing lifestyle-related diseases (%)									
Usually	426	(23.4)	447	(26.2)	280	(11.8)	<0.001	<0.001	
Often	809	(44.5)	702	(41.1)	1248	(52.8)			S-2018 vs. S-2019: 0.647
Rarely	503	(27.6)	448	(26.2)	767	(32.4)			S-2018 vs. S-2020: <0.001
Never	79	(4.3)	107	(6.3)	69	(2.9)			S-2019 vs. S-2020: <0.001
Do not know	6	(0.3)	5	(0.3)	0	(0.0)			
City size of living area									
Big cities	90	(4.9)	93	(5.4)	153	(6.5)	0.409	0.136	
Medium-sized cities (cities with a population of 200,000 or more)	373	(20.5)	362	(21.2)	489	(20.7)			
Medium-sized cities (cities with a population of 100,000 or more)	728	(39.9)	698	(40.8)	970	(41.0)			
Small cities (cities with less than 100,000 inhabitants)	455	(25.0)	392	(22.9)	529	(22.4)			
Towns and villages	177	(9.7)	164	(9.6)	223	(9.4)			

*P*1: χ^2^ test; *P*2: Kruskal-Wallis test; *P*3: Bonferroni’s multiple comparison test results

Nonparametric test results showed that the difference analysis of S-2018, S-2019, and S-2020 had statistical significance, with different internal differences (Table 1). The distributions of SRH, SES, and healthy behaviors in S-2020 significantly differed from those in S-2018 and S-2019. There was no significant difference in SRH, SES, and healthy behaviors between S-2018 and S-2019. The results indicated that the distributions of SRH, SES, and healthy behaviors became worse in 2020.

Table 2 shows the results of binomial logistic regression analyses of the factors associated with SRH. For binomial logistic regression analyses, SRH and SES were divided into two groups, “good/fairly good/neither good nor bad” and “fairly bad/bad,” and healthy behavior was divided into two groups, “always/often” and “rarely/never.” SES was significantly associated with SRH in S-2018, S-2019, and S-2020 after adjusting for age, gender, and resident city size. Adjusted OR was highest in S-2020, indicating that the association between SRH and SES became stronger during COVID-19 outbreak. The association between SRH and healthy behavior was significant in S-2018, S-2019, and S-2020 after adjusting for age, gender, and resident city size. Adjusted OR was highest in S-2020, suggesting that the association between SRH and healthy behaviors became remarkable during COVID-19 outbreak. In other words, people with healthy behaviors tended to keep good SRH during COVID-19 outbreak.

**Table 2.** The Results from Multivariable Analysis.

		Self-rated health (SRH)						
Variable/category		Good/fairly good/ neither good nor bad	Fairly bad/bad	Crude OR	95% CI	Adjusted OR	95% CI	p-value
Subjective economic status (SES)								
S-2018	Good/fairly good/neither good nor bad	1319 (72.4%)	159 (8.7%)	3.11	2.33-4.15	3.09	2.30-4.15	<0.001
	Fairly bad/bad	251 (13.8%)	94 (5.2%)	1.00		1.00		
S-2019	Good/fairly good/neither good nor poor	1203 (70.4%)	140 (8.2%)	2.93	2.18-3.93	2.84	2.11-3.83	<0.001
	Fairly bad/bad	273 (16.0%)	93 (5.4%)	1.00		1.00		
S-2020	Good/fairly good/neither good nor poor	1505 (63.7%)	202 (8.5%)	4.12	3.32-5.12	4.01	3.17-4.93	<0.001
	Fairly bad/bad	423 (17.9%)	234 (9.9%)	1.00		1.00		
Healthy behaviors for preventing lifestyle-related diseases								
S-2018	Always/often	1080 (59.4%)	155 (8.5%)	1.40	1.06-1.83	1.56	1.16-2.10	0.003
	Rarely/never	485 (26.7%)	97 (5.3%)	1.00		1.00		
S-2019	Always/often	1004 (58.9%)	145 (8.5%)	1.29	0.97-1.72	1.39	1.02-1.89	0.035
	Rarely/never	468 (27.5%)	87 (5.1%)	1.00		1.00		
S-2020	Always/often	1301 (55.0%)	227 (9.6%)	1.91	1.55-2.36	2.01	1.56-2.48	<0.001
	Rarely/never	627 (26.5%)	209 (8.8%)	1.00		1.00		

Adjusted model: age, gender, and city size of living area

## Discussion

This study examined the factors related to SRH before and during the spread of COVID-19. SRH became worse during COVID-19 outbreak (S-2020) with higher association with SES and healthy behavior compared with before the outbreak (S-2018 and S-2019). However, the main limitation of this study was that we used individual data from several cross-sectional surveys independently conducted before and during COVID-19 outbreak to measure the effects of the outbreak on SRH. Since the data used in the analysis were not longitudinal, but pooled data from each survey year, we could not reveal real causal relationship.

The association between health inequality and socioeconomic status is well documented across various context and population ^(6), (7)^. Japan currently shows one of the highest levels of average life expectancy in the world, as well as comparatively low levels of health inequality than other developed nations ^(8), (9)^. However, warning bells are sounding about health inequality due to economic inequality in Japan recently ^(10)^. It is suggested that people with bad SES due to financial and job losses are more vulnerable to the COVID-19 outbreak ^(11)^. Holding constant COVID-19-related stress and background controls at both individual and contextual (country) levels, higher income is positively associated with better subjective health ^(12)^. Under unprecedented situations, such as the COVID-19 outbreak, healthy behaviors based on higher health literacy could be helpful in achieving good SRH even though SES condition is relatively poor. It is necessary to consider strategies to promote health education to improve public health outcomes.

## Article Information

### Conflicts of Interest

None

### Sources of Funding

This work was supported by JSPS KAKENHI grant number JP22K02139.

### Acknowledgement

The data for this secondary analysis, “Surveys of Attitudes toward Food and Nutrition Education,” conducted by the Cabinet Office and the Ministry of Agriculture, Forestry and Fisheries of Japan in 2018, 2019, and 2020 were provided by the Social Science Japan Data Archive, Center for Social Research and Data Archives, Institute of Social Science, The University of Tokyo.

### Author Contributions

HI: data analysis and manuscript writing; YI: project development, data management, data analysis, and manuscript writing/editing.
